# Supplementary material for: Consumers Associate High-Quality (Fine) Wines with Complexity, Persistence, and Unpleasant Emotional Responses
Source: Foods. 2020 Apr 8;9(4):452. doi: 10.3390/foods9040452 (PMC7230440; doi:10.3390/foods9040452)
Supplement: Supplementary file 1 [file foods-09-00452-s001.pdf]

Table 1S. Coefficients of correlation (Pearson) found between the variables of the Optimized Descriptive Profile (ODP).

Variables	Smell intensity	Smell complexity	Mouth Thermal perception	Body	Astringency	Persistence	Evolution of the wine in the glass
Smell complexity	0.016	1					
Mouth thermal perception	0.445	**0.892**	1				
Body	0.013	0.812	0.731	1			
Astringency	0.348	0.826	**0.928**	0.855	1		
Persistence	-0.139	0.692	0.559	**0.974**	0.741	1	
Evolution of the wine in the glass	0.011	**0.996**	0.876	0.775	0.780	0.650	1
Duration of the wine fragrance	0.518	0.843	**0.946**	0.664	0.796	0.485	0.852

Note: Values in bold are different from 0 with a significance level $\alpha = 0.05$.

Table 2S. Coefficients of correlation (Pearson) found between the variables (attributes) of the emotional tasting sheet.

Variables	Color	Initial Impression	Smell Intensity	Complexity	Expectation for the mouth	Impression in relation to odor	Thermal perception	Body	Astringency	Persistence	Overall taste evaluation	Evolution of the wine in the glass	Duration of the wine fragrance
Initial Impression	**0.908**												
Smell Intensity	-0.292	-0.488											
Complexity	-0.626	**-0.880**	0.763										
Expectation for the mouth	**0.927**	**0.990**	-0.362	-0.816									
Impression in relation to odor	0.820	0.864	-0.680	-0.804	0.805								
Thermal perception	-0.184	-0.534	0.868	0.871	-0.430	-0.533							
Body	-0.281	-0.606	**0.898**	**0.909**	-0.502	-0.626	**0.993**						
Astringency	-0.362	-0.710	0.453	0.863	-0.675	-0.525	0.781	0.781					
Persistence	-0.078	-0.403	0.876	0.763	-0.279	-0.575	**0.928**	**0.932**	0.685				
Overall taste evaluation	0.817	**0.964**	-0.677	**-0.949**	**0.917**	**0.931**	-0.691	-0.761	-0.755	-0.621			
Evolution of the wine in the glass	0.393	0.224	0.736	0.187	0.359	-0.111	0.573	0.551	0.005	0.708	-0.026		
Duration of the wine fragrance	0.104	-0.153	**0.918**	0.558	-0.014	-0.396	0.842	0.835	0.369	**0.911**	-0.393	**0.918**	
Global evaluation	0.853	**0.992**	-0.571	**-0.934**	**0.967**	0.861	-0.639	-0.702	-0.769	-0.505	**0.981**	0.123	-0.263

Notes: Values in bold are significant ($p<0.05$)

Table 3S. Coefficients of correlation (Pearson) determination found between the variables (ODP x Consumers).

Variables	Smell Intensity	Complexity	Thermal perception	Body	Astringency	Persistence	Evolution of the wine in the glass	Duration of the wine fragrance
Color	0.099	0.152	0.058	0.016	0.008	0.029	0.176	0.016
Initial Impression	0.132	0.479	0.236	0.086	0.169	0.066	0.481	0.120
Smell Intensity	0.300	0.690	0.975	0.399	0.821	0.241	0.657	0.861
Complexity	0.015	0.856	0.628	0.475	0.604	0.397	0.807	0.437
Expectation for the mouth	0.221	0.353	0.127	0.041	0.088	0.035	0.355	0.044
Impression in relation to odor	0.001	0.589	0.450	0.064	0.196	0.025	0.659	0.419
Thermal perception	0.034	0.842	0.838	0.830	0.947	0.703	0.747	0.659
Body	0.035	0.894	0.880	0.753	0.921	0.618	0.814	0.703
Astringency	0.200	0.704	0.302	0.657	0.389	0.695	0.648	0.207
Persistence	0.107	0.874	0.886	0.764	0.796	0.621	0.846	0.895
Overall taste evaluation	0.030	0.717	0.472	0.205	0.338	0.151	0.726	0.341
Evolution of the wine in the glass	0.745	0.188	0.546	0.271	0.481	0.158	0.174	0.639
Duration of the wine fragrance	0.439	0.550	0.879	0.548	0.816	0.381	0.511	0.876
Global evaluation	0.097	0.589	0.332	0.167	0.273	0.134	0.576	0.187

Notes: Values in bold are significant ($p<0.05$)

Figure S1. Final emotional tasting sheet and CATA list of attributes produced after the completion and analysis of the focus group discussions and that was further used in the consumer panel.

TASTING SHEET			
**Name:**		Male ( ) /Female ( )	**Agee:**
**SCALE 1 - 2 - 3 - 4 – 5**			**Wine nº:**
You have received a sample of wine. Please OBSERVE, CHECK and PROVE the sample and indicate how much you liked or disliked, using the scales below, which vary from 1 to 5, according to the description of each attribute:			
**Color**			
Color		Dislike (1) to Like (5)	(1) (2) (3) (4) (5)
**Olfactory Evaluation (Nose)**			
Initial Impression		Distaste (1) to Attraction (5)	(1) (2) (3) (4) (5)
Intensity (Odor)		Weak (1) to Strong (5)	(1) (2) (3) (4) (5)
Complexity		Easy (1) to Difficult to describe (5)	(1) (2) (3) (4) (5)
Expectation for the mouth		Low (1) to High (5)	(1) (2) (3) (4) (5)
**Taste Evaluation (Mouth)**			
Impression in relation to odor		*Disappointing (1) to Surprisingly good (5)*	(1) (2) (3) (4) (5)
Taste Perception	*Fresh (1) to Hot (5)*	*Fresh (1) to Hot (5)*	(1) (2) (3) (4) (5)
	Light (1) to Full-bodied (5)	*Light (1) to Full-bodied (5)*	(1) (2) (3) (4) (5)
	Smooth (1) to Rough (5)	*Smooth (1) to Rough (5)*	(1) (2) (3) (4) (5)
Final Perception	*Short (1) to Long (5)*	*Short (1) to Long (5)*	(1) (2) (3) (4) (5)
	Unpleasant (1) to Pleasant (5)	*Unpleasant (1) to Pleasant (5)*	(1) (2) (3) (4) (5)
**Final Olfactory Evaluation**			
Evolution of the wine in the glass		Unchanged (1) to Evolving (5)	(1) (2) (3) (4) (5)
Duration of the wine fragrance		Short (1) to Long (5)	(1) (2) (3) (4) (5)
**Global Evaluation**			
Dislike a lot ( ) Dislike a little ( ) Do not like nor dislike ( ) Like a little ( ) Like a lot ( )			

Below are several emotional terms. Check all the terms that characterize this wine.				
Pleasant ( )	Aggressive ( )	Joyful ( )	Passionate ( )	Desirable ( )
Daring ( )	Warm ( )	Chewable ( )	Disappointing ( )	Unpleasant ( )
Elegant ( )	Amusing ( )	Cloying ( )	Relaxed ( )	Overwhelming ( )
Euphoric ( )	Interesting ( )	Light ( )	Exhilarating ( )	Melancholic ( )
Peaceable ( )	Greedy ( )	Sensual ( )	Surprising ( )	Sickening ( )
